# Patient Perceived Financial Burden in Haematological Malignancies: A Systematic Review

**DOI:** 10.3390/curroncol29060305

**Published:** 2022-05-24

**Authors:** Catriona Parker, Danielle Berkovic, Darshini Ayton, Ella Zomer, Danny Liew, Andrew Wei

**Affiliations:** 1 School of Public Health and Preventive Medicine, Monash University, Melbourne 3004, Australia; danielle.berkovic@monash.edu (D.B.); darshini.ayton@monash.edu (D.A.); ella.zomer@monash.edu (E.Z.); danny.liew@monash.edu (D.L.); 2 Department of Haematology, Alfred Health, Melbourne 3004, Australia; andrew.wei@monash.edu

**Keywords:** haematology, financial burden, economic, leukaemia, lymphoma, multiple myeloma, systematic review

## Abstract

Advances in scientific understanding have led to novel therapies and improved supportive care for many patients with haematological malignancies. However, these new drugs are often costly, only available at centralised health care facilities, require regular specialist reviews and lengthy treatment regimens. This leads to a significant financial burden. Understanding the impact of financial burden on haematological patients is important to appreciate the urgency of alleviating this systemic issue. Method: Eligible studies were identified by systematically searching Medline, PsycINFO, CINAHL and Embase. Self-reported data reported in both quantitative and qualitative studies that described the financial burden for patients with haematological malignancies were included. Quality appraisal of the included studies was undertaken using the Joanna Briggs Institute tools. A narrative synthesis was employed. For quantitative studies, outcomes were extracted, tabulated and categorised to find similarities and differences between the studies. For qualitative studies, quotations, codes and themes were extracted and then clustered. An inductive approach derived qualitative themes. Results: Twenty studies were identified for inclusion. Of the quantitative studies most (83%) employed un-validated researcher-generated measures to assess financial burden. Between 15–59% of patients experienced a financial burden. Out-of-pocket expenditure was frequent for clinical appointments, prescription and non-prescription medication, and travel. Financial burden was associated with a worsening quality of life and living in metropolitan areas, but there was no evidence for impact on survival. Patient-centred experiences from the qualitative inquiry complemented the quantitative findings and five themes were determined: familial or household impact; reliance on others; barriers to care due to cost; and barriers to accessing financial assistance and sources of out-of-pocket expenses. Conclusion: The impacts of financial burden are yet to be fully appreciated in haematological malignancies, exacerbated by the heterogeneous methods employed by researchers. Future work should focus on identifying the long-term ramifications of financial burden for patients and should trial interventions to reduce its prevalence and patient impacts.

## 1. Introduction

Haematological malignancies are a group of biologically and clinically diverse diseases affecting people of all ages, accounting for approximately 9% of all malignancies in Europe and the United States [1]. Treatment varies from watchful waiting to intensive chemotherapy with or without a haematopoietic stem cell transplant [2].

Advances in scientific understanding have led to novel therapies and improved supportive care, which has increased life expectancy and quality of life for many of these patients [2]. However, the increasing use of high-cost novel agents, prolonged treatment durations, regular and ongoing specialist reviews and the centralised nature of care (requiring travel over long distances for some patients) can leave patients with haematological malignancies particularly vulnerable to high healthcare-related costs. Some of these costs comprise direct medical expenses, such as paying for treatments and diagnostic procedures; others are more indirect, such as lost income due to an inability to work. Of note is that there is a myriad of terms used interchangeably with financial burden, including financial toxicity, financial stress, financial hardship, financial distress, economic burden, economic stress, economic hardship, or economic distress [3]. Whatever the term used, the intent is to describe the stress and hardship arising from the financial burden of cancer treatment [4].

The literature indicates that the patients experiencing a financial burden are more likely to be female, of a younger age, from a lower socioeconomic background and to have a more recent diagnosis [3,5,6]. Furthermore, financial burden has been associated with a reduced quality of life [7,8,9], lower patient satisfaction [10], reduced medication adherence [11,12,13,14] and reduced overall survival [15]. These findings demonstrate the potential complexity and interrelating factors that are associated with financial burden. 

Even though this is known, there remain gaps in the financial burden literature. To date, systematic reviews on the topic have prioritised quantitative studies, have focused on a single country, or have included all cancer types without focusing on the nuances between cancer types. Additionally, many reviews have included insurance database data which, while insightful, potentially exclude uninsured patients and do not reflect the patient perception of their financial burden. There have also been calls to understand the effects of financial burden by cancer type, given the variabilities between cancer treatments, mode of treatment, cancer trajectory and associated out-of-pocket costs [16,17]. In our era of patient-centred care, we were interested to understand the financial burden from the patient perspective. After checking the international prospective register of systematic reviews (PROSPERO) [18], as well as searching the references of recent literature and systematic reviews on the topic of financial burden, we were unable to locate a synthesis of the literature in patients with haematological malignancies. Therefore, the present review aimed to systematically search, appraise, and synthesise the literature of haematological patient-reported data of their financial burden. Four research questions that guided the review were as follows:

1. How is financial burden assessed?
2. What out-of-pocket costs contribute to financial burden (objective financial burden)?
3. What are the impacts of financial burden (subjective financial burden)?
4. What is the patient experience of financial burden?

## 2. Materials and Methods

### 2.1. Design

**Figure 1:**
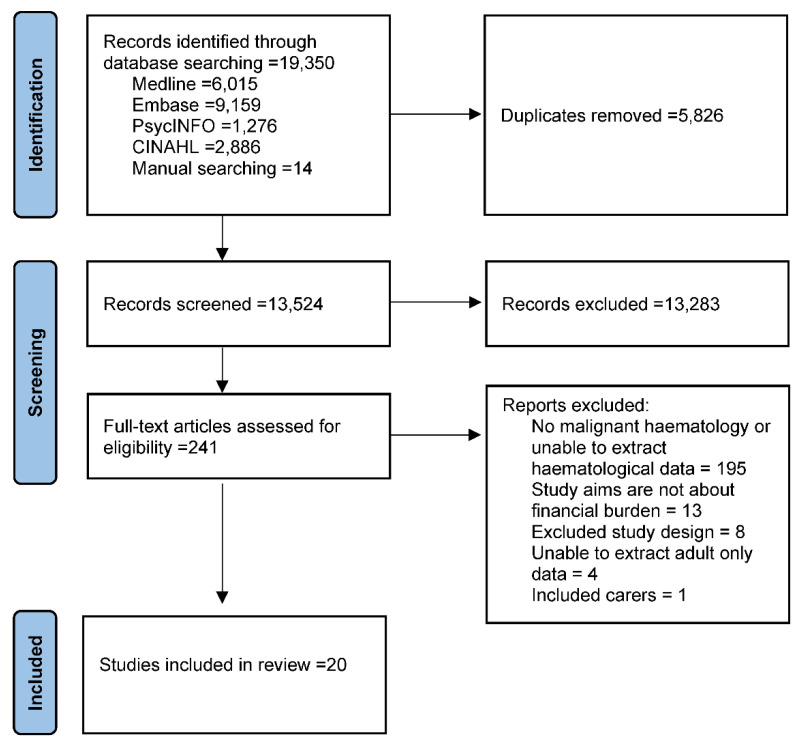
Preferred Reporting Items for Systematic Reviews and Meta-Analysis diagram.

A systematic literature review was undertaken and reported according to the Preferred Reporting Items for Systematic Reviews and Meta-Analysis (PRISMA) statement [19] (Figure 1). The review was registered with the Review Registry (registration number; reviewregistry1361).

### 2.2. Search Strategy

Four electronic databases—Medline, PsycINFO, CINAHL and Embase databases—were searched for English language articles published between 2000 and 2021. The comprehensive search strategy was developed with a specialist librarian and included customised search terms and Boolean operators tailored for each database (see Supplementary Data Files, Tables S1–S3). The search strategy was initially developed for Medline and was then adapted accordingly for other databases. Grey literature, intervention studies or systematic reviews were not included. The reference lists of previously identified key papers, literature, and systematic reviews were manually searched to identify missing additional primary studies in the original search strategy. 

### 2.3. Study Selection

Inclusion criteria of the review included: (a) primary studies of qualitative, quantitative and mixed-method designs; (b) studies that investigated or described the impact of financial burden with data of financial burden coming from the patient directly in one or more haematological malignancies; and (c) patients were aged 18 years and older to ensure that their views or experiences were captured. 

We excluded studies if: (a) patients aged under 18 years were included, and data could not be separated from the rest of the cohort; (b) malignant and non-malignant haematology data were pooled or included all cancer types, and the malignant haematology data could not be separated; (c) the population included carers’ perspectives which could not be separated from those of patients; (d) financial burden was not directly reported by patients or was an incidental finding, rather than the focus of the study; and (e) studies were interventional, randomised controlled trials, systematic reviews or were published as a conference abstract, thesis or book. 

Titles and abstracts were screened by two reviewers (CP and DB) independently using Covidence software (Veritas Health Innovation Ltd., Melbourne, Australia) to determine which studies met the inclusion criteria. The full texts of studies meeting the inclusion criteria were reviewed independently (CP and DB), and reference lists were checked for potentially relevant studies. Any discordance regarding eligibility was discussed and resolved through a consensus between the reviewers.

### 2.4. Data Extraction

Data were extracted from the full-text studies by two independent researchers (DB and CP). A custom template was developed for data extraction, which included the following variables: country of origin, study design, sex, age of participants, haematological malignancy, time of data collection relative to disease, financial burden definition used, financial burden measures and relevant financial impact outcomes (qualitative and quantitative specific). 

### 2.5. Quality and Risk-of-Bias Assessment

The risk of bias was assessed for all included studies by two authors (DB and CP) using the appropriate standardised critical appraisal tools relevant to the study design from the Joanna Briggs Institute (JBI) [20]. The critical appraisal tools ranged from 8 to 10 items, with different questions used to assess the risk of bias depending on the study design. The items were summed, and scores were converted to percentages to compare the quality of evidence scores across different study designs (Supplementary Files, Tables S1–S3). Studies were of low quality if they had a score <50% (and were excluded); of a moderate quality if they had a score between 51–70%, and good-quality studies had a score of 71–100% [21]. None of the included studies were excluded due to a low score. Where there was disagreement between the two reviewers of ±10% on a score, then a consensus was reached through discussion. 

### 2.6. Data Synthesis

Due to the heterogeneity of the studies, a meta-analysis was not undertaken; instead, a narrative synthesis was employed, using elements of the framework described by Popay et al. [22] For the studies reporting quantitative results, characteristics and demographic data such as sample size, gender and age of participants were reported. Then outcomes were extracted, tabulated and categorised to find similarities and differences between the studies. For studies that included other cancer types in addition to haematological malignancies, only the data on haematological malignancies were extracted.

Only those where quotations could be identified for malignant haematological conditions were extracted for qualitative studies that reported cancer-types other than haematological malignancies. The qualitative study narrative synthesis included studies that primarily reported quantitative results and narratives from a free-text field. Quotations and codes used to categorise the data (if available), themes, and other content related to the systematic review were extracted. These data were then organised into categories and were clustered to identify similarities in the data. An inductive approach derived themes which further allowed for relationships between the themes to be explored. 

## 3. Results

### 3.1. Study Selection and Inclusion

After removing duplicates, the database searches yielded 13,524 studies that were screened, and 241 full-text papers were assessed. Finally, 20 studies met the inclusion criteria and were assessed for quality and risk of bias (see Figure 1 for the PRISMA flowchart). No study was excluded after using the JBI quality of evidence assessment tools (see Supplementary Data, Tables S1–S3).

### 3.2. Study Characteristics

The included studies described many impacts of the financial burden arising from haematological malignancies. The studies were undertaken in various countries but predominantly in the USA: USA (12) [23,24,25,26,27,28,29,30,31,32,33,34], Australia (5) [35,36,37,38,39], Canada (1) [40], China (1) [41] and Malaysia (1) [42]. Eleven of the twelve quantitative studies adopted a cross-sectional design [23,24,25,26,27,28,29,30,33,34,39] (Table 1), with the remainder being a cohort study [31]. 

Four of the Australian studies analysed the same participant interviews with different research questions. Of the nine qualitative studies, six of the collected data sets used interviews [32,35,36,37,38,42], one used focus groups [41] and another used both interview and focus group data-collection techniques [40] (Table 2). One quantitative paper was included in the qualitative synthesis as it collected patients narratives but used an open-ended question on a survey [28]. The included papers (both quantitative and qualitative) were published from 2013–2021.

Patients who had received a haematopoietic stem cell transplant (HSCT) between 5 and 36 months prior were the focus of four studies [23,24,29,31]. Four studies recruited multiple myeloma (MM) patients only [28,30,34,40], while two studies focused on patients with chronic myelocytic leukaemia (CML) [26,42]. Eleven studies [23,24,27,29,31,32,35,36,37,38,39] had a mixed malignant haematology cohort. The financial impact of acute myeloid leukaemia (AML) was reported in one study [25]; another included patients with leukaemia [41] (type unspecified) or lymphoma [33] (also type unspecified). Included studies had an age range of 18 to 98 years, and the mean percentage of the participants who were female was 51%. 

### 3.3. RQ1: How Was Financial Burden Assessed?

Ten of the quantitative studies utilised author-derived questionnaires assessing financial burden for which we could not identify validation data [23,24,26,27,28,29,31,33,34,39]. Three of these studies utilised the same 43-item instrument that included financial hardship, household income and employment status [23,24,31]. This instrument came from the literature [43], yet to the best of our knowledge remains un-validated in this population. Another study used a single question from the National Health Interview Survey (NHIS) to measure financial burden on a four-point scale [27]. Hamilton et al. utilised a 9-item researcher-derived questionnaire focusing on financial hardship in which a summative standardised (and linearly transformed) higher score indicated greater financial stress. Another study also used a 32-item researcher-derived questionnaire which was summarised using two composite scores [26]. The first composite included two items on depleted savings or borrowing money, which was defined as financial burden. The second composite score was deemed an indicator of the short-term financial burden and was composed of two questions where respondents indicated they had reduced grocery expenses and utilised co-payment assistance programs. Gupta et al. asked two questions to quantify an estimate of out-of-pocket costs (one question for expenses relating to doctors and another question related to expenses for pharmacy, transport etc.) [34] This study additionally used a Likert scale to understand how overwhelmed patients were by out-of-pocket costs related to their disease. Another study developed a questionnaire comprising of five domains: employment, disability, insurance, retirement and out-of-pocket expenses relating to treatment [28]. Paul et al. used a series of yes/no questions about whether cost influenced treatment decisions, if there was any difficulty paying day-to-day expenses, bills or other payments and if respondents had drawn financial savings [39]. Another study utilised two research-derived questions about whether medical care had been delayed due to cost in the previous 12 months and if respondents needed, but could not afford, medical care in the previous 12 months [33]. 

Two other quantitative studies utilised the validated Comprehensive score for financial toxicity—patient-reported outcome measure (COST-PROM) [25,30]. This brief instrument consists of 11 questions, concerned with satisfaction with finances and income; expenses and the ability to meet them; and the level of control concerning finances and cancer care. The instrument provides a summative score between 0–44, where a lower score indicates a greater financial burden. 

### 3.4. RQ2: What Out-of-Pocket Costs Contribute to Financial Burden?

Two cross-sectional studies reported monetary estimates of out-of-pocket expenses for multiple myeloma [34] and chronic myelocytic leukaemia [26]. Both of these studies were undertaken in the United States. Patients with multiple myeloma in the previous three months spent US$318.90 (±637.20) on clinical appointments, US$388 (±1063.40) for prescription medications, US$191.40 (±363.80) on over-the-counter medications and US$67.30 (±114.80) on transport related to their disease. In total, respondents were US$709 (±1307.30) out-of-pocket for expenses related to their multiple myeloma in the prior three months [34]. More than one-third of the sample (34.6%) reported their financial burden to be high to extremely high due to out-of-pocket costs.

Buzaglo et al. categorised out-of-pocket expenses as at least US$100 per month (49% of respondents), ≥$250 per month (27% of respondents), ≥$500 per month ≥ (16% of respondents) and 6% of respondents were US$1000 per month out-of-pocket [26]. They also found those receiving chemotherapy to have reported a higher mean percentage of income for out-of-pocket expenses during the first 12 months of therapy compared with those not receiving chemotherapy (*p* = 0.025, 95%CI 0.823–12.443).

A final study took a different approach by reporting out-of-pocket expenses as a percentage of income [28]. They estimated a mean percentage of income used on treatment-related expenses to be 36% in the first 12 months after diagnosis and 28% in the preceding 12 months. Additionally, patients receiving chemotherapy had a higher percentage of income used for out-of-pocket costs (*p* = 0.025) and experienced a greater financial burden (*p* = 0.000) [28]. 

### 3.5. RQ3: What Are the Reported Impacts of Financial Burden?

The prevalence of financial burden in haematological malignancies ranged from 15% to 59% across all studies, but importantly, the study designs were primarily cross-sectional, producing low levels of evidence for the impacts of financial burden, with most studies utilising different measures and outcomes. 

After adjusting for sociodemographic factors and significant findings in the bivariate analyses, there was agreement that quality of life was generally worse for patients experiencing financial burden in four studies [23,24,27,29]. There was a reduced overall quality of life demonstrated in three studies [23,24,27]. However, it should be noted that Albelda et al. [24] used a subset of the data reported in Abel et al. [23] to report on employed patients at the point of HSCT. All studies adjusted for age, gender, ethnicity, income and education level. One study additionally adjusted for employment status [29], another for insurance status [27], while the Abel and Abelda studies additionally adjusted for marital status, time since diagnosis, out-of-pocket expenses and the distance travelled to access treatment [23,24]. 

Worsening perceived stress in those with a worsening financial burden was reported in patients 150 days post-HSCT [23], and in the subset analysis of Albelda et al. [24], perceived stress was lower in those with access to paid leave. One paper found that financial burden was associated with reduced functioning in all quality of life domains—physical well-being, social well-being, emotional well-being and functional well-being [29]. 

There were many ways in which patients coped with financial burdens. Reducing spending on basic goods [30], groceries [26,29] and leisure activities [29,30] was described across three studies [26,29,30], with up to 55% of patients implementing one or more of these coping mechanisms. Other ways to financially cope were reported to be depleting savings (range 16–46% of patients) [26,30,39], borrowing money (approximately 20% of patients in both studies) [26,30] and filing for bankruptcy (6% of patients) [26]. The coping mechanisms of depleting savings and borrowing money reached significance (*p* < 0.0001) [30]. One study which analysed a mixed malignant haematology cohort found that living in a metropolitan area had a higher level of financial burden compared with patients living in non-metropolitan areas (*p* = 0.014) [39].

Delaying medical appointments (23% of patients), missing medications (19% of patients) or postponing filling prescriptions (14%) were methods employed by patients with CML to reduce their expenditure [26]. Similarly, Huntington et al. reported that 17% of patients delayed their treatment of multiple myeloma due to cost [30]. Cost factored into decisions about filling only part of a prescription for multiple myeloma treatment (*p* = 0.0077), stopping multiple myeloma treatment (*p* = 0.0011), refusing a test (*p* = 0.016) and skipping a clinic visit (*p* = 0.027) [30]. No confidence intervals were reported. Jella et al. reported lymphoma patients who delayed medical care due to cost were more than twice as likely to self-report their health status as poor to fair compared with good to excellent (OR: 2.47, 1.59–3.83, *p* < 0.001), as were patients who needed medical care but could not afford it in the past 12 months (OR: 2.08, 1.23–3.49, *p* = 0.006) [33].

Using a medication adherence score, Gupta et al. found a lower adherence to medication with higher financial burden for over-the-counter medications (*p* = 0.006) and transportation (*p* = 0.03). This association did not reach a significance of *p* < 0.05 for cost of clinical appointments, prescription medications or total out-of-pocket expenses (no *p*-values provided) [34].

A single prospective cohort study of 325 patients investigated the impact of financial burden on survival 1–2 years after HSCT [31]. There was no significant difference in survival between those suffering financial burden and those that did not [31].

### 3.6. RQ4: What Is the Patient Experience of Financial Burden?

Five main themes arose from nine qualitative studies [28,32,35,36,37,38,40,41,42] reflecting the patient experience of the impact of financial burden (see Supplementary Data for additional supporting quotations, Tables S1–S3): the familial or household impact; relying on others for financial support; barriers to care due to cost; barriers to gaining financial assistance; and sources of out-of-pocket expenses. 

The familial or household impacts of financial burden described individual perceptions or feelings, financial coping mechanisms, making everyday choices and the long term ramifications of financial decisions. Emotionally, the burden of financial worries manifested in patients in different ways “...*the financial impact... is really stressful*” [40] while others felt a “*…feeling of inadequacy and not being to provide for the family and the heaviness that I felt all the time*” [32].

Patients discussed the reality of how the burden of financial impact changed how they lived their everyday lives: “…*we started living like we did back when our kids were little, and we did not have any money*” [32]. Being more budget-conscious was a common financial coping mechanism such as “.*..watching my* spending *a lot more*” [32] *and being* “*…mindful of where every penny was going*” [32]. One patient explained, “*[the cost of] medicine was high—sometimes you have to choose between medicine and food*” [28]. There was evidence that financial burden can have future impacts on a person’s financial health: “*credit card payments—still paying them after eight years, credit suffered, sold [our] home, borrowed money from relatives*” [28]. Another patient said, “*we lost everything, including our home*” [28].

At times, patients relied on others for financial assistance, accepting financial help through friends, family and their religious community. One patient explained the necessity of this income stream to stave off financial ruin: “*I have had to rely on gifts from family and friends to keep from filing bankruptcy*” [28]. Another described how utilising the Internet for crowdfunding to raise funds helped them financially: “*…the Go Fund Me is basically what got us through*” [32].

However, for some, their financial burden was significant enough to impact the decisions surrounding their medical care, where cost represented a barrier to care: “*I quit doing it (photopheresis) because it was expensive and we are trying to find something more cost-effective*” [32]. Patients also described barriers to gaining financial assistance through services designed to financially aid patients. One patient explained how she had to go to great lengths to obtain approval for financial reimbursement: “*I had to make myself look like a madwoman, messing up my hair, thumping my prescription for morphine injections and my medical certificate showing I had advanced-stage cancer down on the official’s desk; only then would he sign the approval*” [41]. Another described how one government body requires “…*you use up all your money and have no money in the bank*..” [35] before granting financial assistance “*which is ridiculous as you don’t have money to fall back on. Because that is the money you have saved up for your registration and your rates (bills)*” [35]. One patient concluded that “*These officials were just inconsiderate and unsympathetic*” [41].

An additional descriptive qualitative study [36] included quotations (see Supplementary Data, Tables S1–S3) from participants detailing their sources of out-of-pocket expenditure; costs associated with travel and accommodation (e.g., flights, parking, food, tolls and fines); costs associated with the care of family and friends (e.g., childminding expenses, phone bills, paying rent when not at home); and costs associated with diagnosis and treatment (e.g., treatments, pharmacy bills and gap payments). 

## 4. Discussion

This systematic review aimed to provide evidence of the impact of financial toxicity on patients with haematological malignancies. 

We identified that most of the quantitative measures utilised to assess financial burden in the literature for patients with haematological malignancies are researcher-designed questionnaires [23,24,26,27,28,29,31,33,34,39]. In contrast, only two studies utilised a validated PROM (COST-PROM) [25,30], and one final study used a single question from the NHIS [27]. This methodological heterogeneity presents substantial problems in comparing findings across studies. Additionally, this highlights the lack of conceptual clarity of the financial burden experienced by patients [44]. Conceptual models assist in building domains within measurement instruments for the consistent measurement of agreed constructs, representing nuanced and unique factors. Without an agreed conceptual model and consensus language, there is an inconsistent use of theory by research [45]. For the field of financial burden research, this has manifested in varying measurement instruments and most remain unvalidated. In the present study, it is therefore difficult to comment on the validity of results and to confidently generalise the findings.

Altice and colleagues have proposed a typology representing three broad domains that constitute financial hardship: (a) material conditions that arise from increased out-of-pocket expenses and reduced income; (b) the psychological response, such as distress and concern at managing unexpected health-care-related financial expenditure or reduction in income; and (c) coping behaviours which patients adopt to manage their care while experiencing increased expenditure and reduced income [3]. Carrera et al. [46] proposed a conceptual framework of financial toxicity, as did Witte et al. [47] the following year. While these frameworks are helpful, a shared comprehensive conceptual framework is yet to be elucidated, which may be contributing to the heterogeneous methodology employed by researchers. Furthermore, work needs to be undertaken to explore how the emphasis on different components of the frameworks may vary depending on cancer diagnosis (due to differing disease patient demographics, disease trajectories, treatments) and by country (for example, the difference in out-of-pocket costs borne by the patient in user-pays healthcare models compared with countries with publicly funded healthcare). In the interests of collaborative scholarship and to allow for comparisons within and between cancer types, researchers should be encouraged to utilise existing (validated) financial hardship instruments rather than generating their own question sets.

Our review identified three main categories of impact and patient experience from financial burden: (1) reduced quality of life and well-being (for example, emotional well-being); (2) introduction of financial coping mechanisms; and (3) compromised care due to cost. The findings are broadly consistent with the wider financial burden literature where, for example, using a national survey, Yabroff et al. found in the United States medical financial hardship in the past year was particularly common in material (e.g., medical debt), psychological (e.g., worry) and behavioural (e.g., delaying care) domains [48]. Comparing those with and without cancer, those with cancer are more likely to report material, psychological and behavioural hardship compared to a cohort without cancer [49].

The literature identified in this review suggests that patients with haematological malignancies experiencing a financial burden have a reduced quality of life [23,24,27,29] and other well-being impacts [24,29]. However, in the present review, all studies reporting these outcomes were in patients that had undergone HSCT. Therefore, the quality of life outcomes for patients not prescribed this treatment pathway remain unknown. Each study utilised a different quality of life measure (two utilised two questions from the EORT-QLQC30 [23,24] with modified wording, one used the NHIS [27] and another utilised FACT-BMT [29]) and included varying haematological malignancies. While varying measurement instruments were employed across these four studies, the finding of financial burden impacting the quality of life and well-being is congruent with other cancer types [5,8,50]. Patient stress was also shown to increase with the objective financial burden [23]. The qualitative studies add a further context to quality of life findings, where the emotional impact of financial burden was described as being stressful. The findings in this review are congruent with the literature in other cancer types, where there is an established relationship between financial burden and poorer psychosocial outcomes [51,52,53]. Furthermore, when compared with non-cancer sufferers, those with cancer more frequently report stress and worry related to financial troubles [49]. Future work should focus on longitudinal studies to explore the quality of life and other psychosocial outcomes vary throughout financial burden and provide evidence for the most appropriate financial burden measurement time points [54]. 

The financial coping measures employed by patients to reduce usual expenditure in the reviewed studies highlight how patients trade aspects of their comfort and lifestyle to afford expenses related to their healthcare and depleted income [25,26,29,30,39]. The qualitative studies demonstrated how this impacted the wider family and household [32]. Quantitative studies highlighted the prevalence of patients implementing financial coping actions (between 20% and 55%). These findings were complemented by the patient narrative of how these experiences affect the patients’ emotional state while relying on other sources of financial support. Patients described bureaucratic and unsympathetic processes to access insurance payments or government financial support, which may present a barrier to patients accessing their financial entitlements, but this needs to be further investigated. While it may seem insurmountable to change big business and Government processes, there may be other avenues to assist patients in need. For example, in the United Kingdom, a welfare rights advice program designed to address the financial burden from cancer successfully demonstrated positive effects on social and psychological patient outcomes [55]. Similarly, participation in a debt management program (albeit not specific to those with cancer) in the United States showed a similar benefit [56].

The qualitative and quantitative literature were aligned concerning the cost of care impacting patients’ healthcare decisions about their medical care. These decisions included delaying or missing clinical appointments or prescription medication, particularly in patients diagnosed with CML. Patients with CML have clinically benefited in recent years from the new era of tyrosine kinase inhibitors (TKIs)—with medication adherence, these patients can have a life expectancy similar to the general population [57]. However, even with generic pricing, which bought about a reduction in the cost of TKIs, the medication may remain unaffordable [52] for many patients, potentially affecting progression-free and overall survival through poor medication adherence. 

Nevertheless, understanding these coping mechanisms employed by patients will be imperative to designing, testing, and implementing meaningful interventions to alleviate the financial burden and to minimise patient actions that involve sacrificing their health care due to their financial position. Future work should focus on unravelling how patient financial burden alters along the care trajectory in order to ensure that any interventions are targeted and timely. For example, the costs of care for multiple myeloma patients have been shown to vary throughout the care trajectory, with higher costs for the initial treatment phase [58]. However, it will be important to understand how and when this translates to greatest patient financial distress, which foreseeably may vary by treatment regimens and could be dependent on varying health systems and their associated reimbursements or coverage.

Our review contributes the first systematic appraisal of the literature concerning the impact of financial burden in patients with haematological malignancies. Much of the identified evidence has been drawn from the United States, which adopts a user-pays model of health care in contrast with the hybrid and socialised models employed elsewhere in the world. Therefore, the contributing factors to financial burden and the measurable impacts on patients may vary by healthcare model. However, this is poorly understood. Furthermore, even though this review focused on haematological malignancies, this cancer group still represents a very heterogenous patient cohort and variation in the treatment regimens employed. It is difficult therefore to generalise the financial burden findings from one haematological malignancy to the next.

Financial burden by age was not well reported in the identified literature, even though financial burden has been shown to affect younger oncology patients more severely [5]. Haematological malignancies provide a unique opportunity to examine the relationship of financial burden with age (as well as outcomes and interventions), particularly as this disease group uniquely affects people of all ages. The strengths of this review include searching multiple databases with a thorough search strategy to capture the relevant literature over the past twenty years. Nevertheless, it remains possible that relevant research was missed due to the different indexing used by different databases and the inconsistent terminology used to describe financial burden. To minimise this limitation, a manual search of the reference lists was undertaken of the identified reviews during screening, the studies known to the authors, and the included literature in the present study. 

We limited the review to exclude studies without a financial burden focus to ensure the review was manageable, excluding papers with incidental financial burden findings. Furthermore, due to the research questions guiding this review, we excluded papers that only provided a prevalence of financial burden, and as such, the estimate provided in this manuscript should be viewed within that context. Additionally, we did not include changes in employment status and therefore house income changes, which conceivably may impact on the patient perception of financial security. 

## 5. Conclusions

The impacts of financial burden are yet to be fully appreciated in haematological malignancies, and are exacerbated by the heterogeneous methods employed by researchers. Of concern is the financial burden and its association with poor medication adherence to TKIs. Additionally, there was evidence of financial burden for patients undergoing HSCT, which may be due to the protracted illness, treatment and recovery trajectory. Future work should focus on examining how the financial burden alters along the cancer care journey, identifying the long-term ramifications of financial burden for patients and using this information to trial methods to reduce its prevalence and patient impacts. 

## Figures and Tables

**Table 1:** Included quantitative studies.

Author, Year, Country	Study Design	Sample Size	Sample Age Reported as Mean, Median or Range (Years)	Percentage Female (%)	Included Haematological Conditions	Timing of Assessment	Main Findings Describing Financial Impact
Abel et al., 2016, USA [23]	Cross-sectional	325	Median, 61	40	MM, NHL, AML, MDS, HL, ALL, other	150 days post-HSCT	Unsatisfied with present financial situation = 49% of sample Difficulty meeting monthly payments = 42% of sample Not enough money at months-end = 19% of sample Difficulty paying for HSCT-related costs = 51% of sample Difficultly paying for transportation = 41% of sample Difficulty meeting costs of changed home environment = 19% of sample. Income decline = 46% of sample Multivariate analysis of financial hardship measures with patient-reported outcome measures QOL below median Income decline: OR 1.62 (95% CI: 0.98–2.7, *p* = 0.06) Hardship_1: OR 2.9 (95% CI:1.7–4.9, *p* < 0.001) Hardship_2: OR 2.16 (95% CI: 0.99–4.7, *p* = 0.05) Self-reported health below the median Income decline: OR 1.33 (95% CI: 0.81–2.2, *p* = 0.26) Hardship_1: OR 2.18 (95% CI: 1.3–3.6, *p* = 0.003) Hardship_2: OR 1.88 (95% CI: 0.89–3.9, *p* = 0.10) Perceived stress above median Income decline OR: 2.07 (95% CI: 1.3–3.4, *p* = 0.004) Hardship_1: OR 2.08 (95% CI: 1.3–3.5, *p* = 0.005) Hardship_2: OR 3.14 (95% CI: 1.4–6.8, *p* = 0.004)
Albelda et al. *, 2019, USA [24]	Cross-sectional	171	Mean, 57	NR	Any needing BMT, but NR	6-months post HSCT	Multivariate analysis of financial burden with: “Dissatisfied with financial situation” (OLS coefficients, 95% CI) Health: −0.331, (−0.501, −0.161), *p* < 0.01 Quality of life: −0.295, (−0.473, −0.118), *p* < 0.01 Perceived stress: −1.093, (−1.496,−0.689), *p* < 0.01 “Difficulty paying bills” (OLS coefficients, 95% CI) Health: −0.270 (0.433,−0.108), *p* < 0.01 Quality of life: −0.177 (−0.348, 0.006), *p* < 0.05 Perceived stress: −0.720 (−1.118,−0.321), *p* < 0.01 “Not enough money at the end of the month” (OLS coefficients, 95% CI) Health: 0.404 (−0.680,−0.128), *p* < 0.01 Quality of life: −0.321 (−0.601, −0.024), *p* < 0.05 Perceived stress: −0.943 (−1.625, −0.261), *p* < 0.01
Bala-Hampton et al., 2017, USA [25]	Cross-sectional	26	Mean, 58.5 (SD 14.1)	46.2	AML	6 months after diagnosis	Not enough money to cover the cost of treatments = 69.2% of the sample Out-of-pocket expenses greater than expected = 65.4% of the sample Increased financial worry = 77% of the sample No choice in the cost of the care = 85% of the sample Unable to financially contribute to the household = 62% of the sample Dissatisfaction with finances = 73% of the sample Felt financially stressed = 69.2% of the sample Felt not in control of their finances = 85% of the sample
Buzaglo et al., 2017, USA [26]	Cross-sectional	318	Mean, 56 Range, 18–85	68	CML	Mean of 5.2 years from diagnosis	Out of pocket costs (%of the sample) Spent at least US$100 per month = 49% Spent ≥ US$250 per month = 27% Spent ≥ US$500 per month = 16% Spent ≥ US$1,000 per month = 6% To reduce the cost associated with CML (% of sample): Postponed seeking psychological counselling (sometimes, often, or always) = 23% Missing a dose or oral CML drugs at least monthly = 19% Delayed follow-up on recommendations on complementary treatment = 17% Postponed doctor’s appointments = 16% Postponed filling prescriptions = 14% Skipped doses or CML oral drugs at least sometimes = 10% Because of costs associated with CML (% of sample which varied from 283–287 respondents): Reduced grocery expenditure = 35% Depleted savings = 33% Borrowed against or used money from retirement = 20% of sample Sold personal property = 18% Liquidated assets = 13% Refinanced house = 8% Filed for bankruptcy = 6% Home foreclosed = 4% Multivariate analysis with financial burden Suboptimal treatment adherence *p* < 0.001
Fenn et al., 2014, USA [27]	Cross-sectional	NR for haematology	NR for haematology	NR for haematology	leukaemia/lymphoma	NR	Multivariate analysis with financial burden and QoL of at least ‘good’ Adjusted OR = 0.91, 95% CI 0.42–1.95, *p* = 0.799
Goodwin, et al., 2013, USA [28]	Cross-sectional	762	Mean, 61 (SD 9.26)	39	MM	Received intensive treatment at the site	Out-of-pocket costs as a percentage of income by time since treatment began % income spent during first year of treatment Treatment began < 4years ago = 40% Treatment began ≥ 4 years ago = 33% t = −2.281, *p* = 0.023, 95%CI −13.658–1.019 % income spent in past 12 months Treatment began < 4years ago = 35% Treatment began ≥ 4 years ago = 23% t = −5.465, *p = 0*.0005, 95%CI −16.921–7.968 Out-of-pocket costs as a percentage of income by time since treatment ended % income spent during first year of treatment Treatment ended < 4years ago = 37% Treatment began ≥ 4 years ago = 37% t = −0.14, *p* = 0.998, 95%CI −11.015–10.854 % income spent in past 12 months Treatment ended < 4years ago = 29% Treatment began ≥ 4 years ago = 22% t= −2.143, *p* = 0.033, 95%CI −13.21–0.564 Other findings Percentage of income used for out-of-pocket costs Mean percentage of income used on treatment-related expenses = 36% during the first 12 months Mean percentage of income used on treatment-related expenses = 28% in the past 12 months Treatment costs are somewhat to very much a burden to themselves or family = 42% of the sample. Income use by treatment modality Percentage of income used for those on chemotherapy vs. not t = 2.03, *p* = 0.025, 95% CI 0.823–12.443 ingle item from the FACT-BMT regarding burden of treatment costs Financial burden for patients on chemotherapy treatments vs. not t= −3.51, *p* = 0.000, 95% CI: − 0.57 to − 0.16
Gupta et al., 2018, USA [34]	Cross-sectional	162	Mean, 55.9 (SD 13.5)	49.4	MM	First line treatment: medicated for ≥8 weeks Second line treatment: ≥6 weeks	Out-of-pocket costs (US$) Cost of clinical appointments = $318.90 (±637.20) Prescription medications = $388 (±1063.40) Over the counter medications = $191.40 (±363.80) Transportation = $67.30 (±114.80) Total out-of-pocket = $709 (±1307.30) Financial burden related to out-of-pocket costs (n, %) None = 48 (29.6) Some = 46 (28.4) Moderate = 50 (30.9) High = 28 (17.3) Extremely high = 7 (4.3) ** MMAS, out-of-pocket costs and financial burden generalised linear modeling (adjusted mean ± SE, 95% CI) Cost of clinical appointments Score ≤ 3 = 147.7 ± 45.7, 80.6–270.6, *p* > 0.05 Score 4 = 210.3 ± 49.9, 132.1–334.7 Prescription medications Score ≤ 3 = 387.9 ± 168.4, 165.7–908.1, *p* > 0.05 Score 4 = 220.2 ± 68.4, 119.8–404.8 Over the counter medications Score ≤ 3 = 130.6 ± 34.0, 78.3–217.6, *p* = 0.006 Score 4 = 46.8 ± 9.1, 32.0–68.4 Transportation Score ≤ 3 = 83.0 ± 18.6, 53.5–128.8, *p* = 0.03 Score 4 = 43.3 ± 7.6, 30.6–61.2 Total out-of-pocket Score ≤ 3 = 828.3 ± 248.7, 459.9–1491.8, *p* > 0.05 Score 4 = 395.7 ± 87.2, 256.8–609.5 Financial burden related to out-of-pocket costs by MMAS (adjusted mean ± SE, 95% CI) Score ≤ 3 = 0.7 ± 0.1, 0.6–0.9, *p* > 0.05 Score 4 = 0.6 ± 0.1, 0.5–0.8
Hamilton et al., 2013, USA [29]	Cross-sectional	181	NR	55.2	Eligibility: any haematological malignancy requiring HSCT Sample: NR (participants were required to be at least moderately distressed according to standardised measure delivered pre-study)	9–36 months post HSCT	Perceptions of economic survivorship stressors: Sources of financial stress occurred most frequently as ‘moderately’ or ‘a great deal’ in the past month, including (% of the sample): Reducing or cancelling vacations or leisure activities = 34% Reducing spending on household expenses such as food or clothing = 33% Deciding not to buy something they had planned to purchase = 28% Difficult, very difficult, or extremely difficult to live on their income = 23% Anticipated reducing their standard of living to afford the bare necessities in life ‘at least somewhat’ = 22% Hierarchical regression of financial stress and HRQoL (reported F change, significance) Physical wellbeing −4.05 *p* < 0.001 Social wellbeing −1.03, *p* > 0.05 Emotional wellbeing −3.36, *p* < 0.001 Functional wellbeing −2.83, *p* < 0.01
Huntington et al., 2015, USA [30]	Cross-sectional	100	Mean = 64.1 (SD 9.8) Median = 64.7 (Range: 38.4–90.2)	53	Multiple myeloma	3 months after treatment commenced	55/100 patients reported reduced spending on basic goods 6/98 patients reported reduced spending on leisure 43/94 patients used savings to pay for treatment 21/98 patients borrowed money 17/100 reported delays in treatment of their multiple myeloma because of cost 36/100 patients applied for financial co-payment assistance 59/100 reported out-of-pocket treatment costs for MM were higher than expected Decreased spending on basic goods (food and clothing): *p* < 0.0001 Decreased spending on leisure activities: *p* < 0.0001 Use savings to pay for cancer care: *p* < 0.0001 Borrow money for cancer care: *p* < 0.0001 Delay the start of a myeloma treatment: *p* = 0.0030 Fill only part of myeloma therapy prescription because of cost: *p* = 0.0077 Stop myeloma therapy prescription because of cost: *p* = 0.0011 Refuse recommended test because of cost: *p* = 0.016 Skip clinic visit to save on costs: *p* = 0.027 Apply for financial assistance: *p* = 0.14
Jella et al., 2021 USA [33]	Cross-sectional (collected annually between 1997–2018)	1619	NR	47	Lymphoma	NR	Medical care delayed due to cost, past 12 months? Yes = 161 (10%) No = 1458 (90%) Needed but could not afford medical care, past 12 months? Yes = 105 (7%) No = 1513 (93%) Multivariate analysis of financial stressors (adjusted odds ratio, 95%CI, *p* value) Medical care delayed due to cost, past 12 months? Age (years) 18–24 = 0.87 (0.15–5.09), *p* = 0.881 25–44 = 4.63 (2.28–9.41), *p* < 0.001 45–64 = 5.85 (3.20–10.70), *p* < 0.001 ≥65 = Reference Race/ethnicity White = Reference Black = 0.89 (0.44–1.84), *p* = 0.760 Hispanic = 1.63 (0.73–3.65), *p* = 0.237 Other = 1.08 (0.49–2.36), *p* = 0.845 Sex Male = Reference Female= 1.62 (1.06–2.48), *p* = 0.027 Born in the United States Yes = Reference No = 0.27 (0.09–0.83), *p* = 0.024 Marital Status Married = Reference Single = 1.88 (1.18–3.00), *p* = 0.009 Self-reported Health status Good to excellent = Reference Poor to fair = 2.47 (1.59–3.83), *p* < 0.001 Needed but could not afford medical care, past 12 months? Age (years) 18–24 = 0.23 (0.17–1.07), *p* = 0.172 25–44 = 3.50 (1.13–8.24),*p* = 0.004 45–64 = 4.87 (2.33–10.17), *p* ≤ 0.001 ≥65 = Reference Race/ethnicity White = Reference Black = 0.81 (0.35–1.88), *p* = 0.620 Hispanic = 0.42 (0.17–1.07), *p* = 0.070 Other = 1.71 (0.69–4.23), *p* = 0.247 Sex Male = Reference Female = 2.20 (1.28–3.76), *p* = 0.004 Born in the United States Yes = Reference No = 0.14 (0.02–0.88), *p* = 0.037 Marital Status Married = Reference Single = 1.63 (0.93–2.85), *p*= 0.087 Self-reported Health status Good to excellent = Reference Poor to fair = 2.08 (1.23–3.49), *p* = 0.006
Khera et al. ***, 2018, USA [31]	Cohort	325	NR	40	MM, NHL, AML, MDS, HL, ALL, other	1 and 2 years survival, post HSCT	Univariate analysis (Hazard Ratio (95% CI)) Hardship No N = 141 1-year survival HR 0.96 (0.92–0.98) 2-year survival HR 0.91 (0.85–0.95) Yes N = 182 1-year survival HR 0.94 (0.89–0.97) 2-year survival HR 0.87 (0.81–0.91) Extreme Hardship No N =273 1-year survival 0.94 (0.91–0.96) 2-year survival 0.89 (0.84–0.92) Yes N= 50 1-year survival HR 1.00 (-) 2-year survival HR 0.92 (0.79–0.97)
Paul, et al., 2013, Australia [39]	Cross-sectional	268	Mean = 59.5 (SD 13.4)	41	NHL, lymphoma, leukaemia, MM	Diagnosed in the previous 3 years	Difficulty paying bills of other payments (% of the sample by participants residing in metropolitan or non-metropolitan areas) Metropolitan= 24% Non-metropolitan = 16% Χ^2^ =2.56, *p* = 0.11 Used up savings (% of the sample by participants residing in metropolitan or non-metropolitan areas) Metropolitan =25% Non-metropolitan = 16% Χ^2^ = 2.98, *p* = 0.084 Had trouble with day-to-day expenses (% of the sample by participants residing in metropolitan or non-metropolitan areas) Metropolitan = 15% Non-metropolitan = 8% Χ^2^ = 3.55, *p* = 0.06 Other findings (% of the total sample) Cancer-related expenses influenced decision about treatment = 2% Cancer-related out-of-pocket expense = 45% of the sample Percentage of respondents with out of pocket expenses relating to: - parking for medical appointments = 33% - travel costs to appointments = 30% - treatment drugs = 24% - assistance with gardening or housework = 8% - other medical supplies = 4.6% - accommodation while at appointments = 2.3% Difference between metropolitan and non-metropolitan out-of-pocket expenses = F(1,260)= 0.40, *p* = 0.528 Financial burden from living in a metropolitan city vs. non-metropolitan = χ^2^ =6.06, *p* = 0.014

ALL = acute lymphoblastic lymphoma, AML = acute myeloid leukaemia, CI = confidence interval, CML = chronic lymphocytic leukaemia, FACT-BMT = Functional Assessment of Cancer Therapy—Bone Marrow Transplantation, HL = Hodgkin’s lymphoma, (HR) QoL = (health related) quality of life, HSCT = haematopoietic stem cell transplant, MDS =myelodysplastic syndrome, MM = multiple myeloma, MMAS= Morisky Medication Adherence Scale, MPD = myeloproliferative disorder, NHIS= National Health Interview Survey, NHL = non-Hodgkin’s lymphoma, NR = not reported, OR = odds ratio, SD = standard deviation, USA = United States of America. * This study is a sub-set analysis of the original data collection by Abel [23]. ** MMAS 0–4 scale where higher scores represent greater adherence. *** This study used the original cohort from Abel et al. [23].

**Table 2:** Included qualitative studies.

Author, Year, Country	Age Range of Participants (Years)	%Female	Included Haematological Malignancies	Measurement Time-Point	Study Design	Data Collection Technique	Data Analysis Technique
Goodwin et al., 2013, USA [28]	29–77	39	MM	Patients had received intensive therapy (between 0–42 years prior)	Cross-sectional	Open ended survey question	NR
Head et al., 2018, USA [32]	30–67	77	Any	1–5 years after diagnosis. Participants were experiencing financial hardship as defined by three questions from the COST-PROM	NR	Interviews	Thematic (constructivist grounded-theory approach)
McGrath, 2015, Australia * [38]	18–≥70	56	HL, NHL, AML, ALL, APML, CML, CLL, MM, MDS, MN-ET	NR	Descriptive	Interviews	Thematic
McGrath, 2016, Australia * [35]	18–≥70	56	HL, NHL, AML, ALL, APML, CML, CLL, MM, MDS, MN-ET	NR	Descriptive	Interviews	Thematic
McGrath, 2016, Australia * [36]	18–≥70	56	HL, NHL, AML, ALL, APML, CML, CLL, MM, MDS, MN-ET	NR	Descriptive	Interviews	Thematic
McGrath, 2016, Australia * [37]	18–≥70	56	HL, NHL, AML, ALL, APML, CML, CLL, MM, MDS, MN-ET	NR	Descriptive	Interviews	Thematic
Parsons et al., 2019, Canada [40]	51–83	31	MM	Relapse or refractory disease	Descriptive	Interviews, followed by focus groups	Thematic
Tan et al., 2017, Malaysia [42]	26–67	50	CML	Taking tyrosine kinase inhibitor	NR	Interviews	Thematic
Wang et al., 2016, China [41]	42–78	74	Leukaemia	Cancer survivors	NR	Focus groups	Thematic

* These studies analysed the same participants, ALL = acute lymphoblastic lymphoma, AML = acute myeloid leukaemia, APML = acute pro-myelocytic leukaemia, COST-PROM = Comprehensive score for financial toxicity patient reported outcome measure, CML = chronic myelocytic leukaemia, CLL = chronic lympocytic leukaemia, HL = Hodgkin’s lymphoma, MDS = myelodysplastic syndrome, MM = multiple myeloma, MN-ET = myeloproliferative neoplasm—essential thrombocythemia, NHL = non-Hodgkin’s lymphoma, NR = not reported, USA = United States of America.

## Data Availability

The datasets generated and/or analysed during the current study are available from the corresponding author on reasonable request.

## Supplementary Materials

The following supporting information can be downloaded at: https://www.mdpi.com/article/10.3390/curroncol29060305/s1, Table S1: search strategy; Table S2: quality appraisal scores; Table S3: additional quotations.
