# Supplementary material for: Patient Perceived Financial Burden in Haematological Malignancies: A Systematic Review
Source: Curr Oncol. 2022 May 24;29(6):3807–24. doi: 10.3390/curroncol29060305 (PMC9221876; doi:10.3390/curroncol29060305)
Supplement: Supplementary file 1 [file curroncol-29-00305-s001.zip › curroncol-1665657-supplementary.pdf]

Supplementary file S1: Patient perceived financial burden in haematological malignancies: a systematic review

Search strategy developed for Medline

1. exp neoplasms/
2. (tumo?r* or carcinoma* or metasta* or oncolog* or cancer* or malignan*).mp.
3. 1 OR 2
4. (Family budget or Household budget or household expense* or household expenditure* or household budget or financial budget).mp.
5. (financ* adj2 vulnerab*).mp.
6. (financial toxicity or financial burden or financial distress or financial stress or financial impact or financial worry or financial worries or financial sacrifice*).mp.
7. (economic burden or economic stress or economic distress or income stress or income distress or budget impact or budget stress or budget distress or money stress or money distress or money worries or money worry).mp.
8. hardship.mp.
9. out-of-pocket.mp.
10. (bankrupt* or financial insolvenc* or credit payment* or mortgage payment* or borrowing money).mp.
11. (financ* adj3 treatment*).mp.
12. (financ* adj3 therap*).mp.
13. (medical debt or debt burden or going into debt or mortgage debt or debt stress or debt distress).mp.
14. (medical care cost* or medical treatment cost* or cancer care cost* or cancer treatment cost*).mp.
15. exp "Cost of Illness"/
16. 4 OR 5 OR 6 OR 7 OR 8 OR 9 OR 10 OR 11 OR 12 OR 13 OR 14 OR 15
17. 16 AND 3

18. limit 20 to (english language and yr="2000 -Current")

Supplementary File S2: Patient perceived financial burden in haematological malignancies: a systematic review

Supplementary File: JBI scores

**Author**	**Year**	**Country**	**Study design**	**JBI score**
Abel *et all*[[23]	2016	USA	Cross-sectional	87.5%
Abelda *et al* [24]	2019	USA	Cross-sectional	100%
Bala-Hampton *et al* [25]	2017	USA	Cross-sectional	62.5%
Buzaglo *et al* [26]	2017	USA	Cross-sectional	62.5%
Fenn *et al* [27]	2014	USA	Cross-sectional	75%
*Goodwin *et al* [28]	2013	USA	Cross-sectional	75%
Gupta *et al* [34]	2018	USA	Cross-sectional	87.5%
Hamilton *et al* [29]	2013	USA	Cross-sectional	87.5%
Head *et al* [32]	2018	USA	Qualitative	80%
Huntington *et al* [30]	2015	USA	Cross-sectional	100%
Jella *et al* [33]	2021	USA	Cross-sectional	75%
Khera *et al* [31]	2018	USA	Cohort	75%
McGrath [38]	2015	Australia	Qualitative	70%
McGrath [35]	2016	Australia	Qualitative	70%
McGrath [36]	2016	Australia	Qualitative	70%
McGrath [37]	2016	Australia	Qualitative	70%
Paul *et al* [39]	2013	Australia	Cross-sectional	87.5%
Parsons *et al* [40]	2019	Canada	Qualitative	80%
Tan *et al* [42]	2017	Malaysia	Qualitative	70%
Wang *et al* [41]	2016	China	Qualitative	70%

Supplementary file S3: Patient perceived financial burden in haematological malignancies: a systematic review

Supplementary data: Additional supporting quotations

**Themes**	**Sub-themes**	**Illustrative quotes**
Out-of-pocket expenditure[36]	Travel and accommodation	We were paying double costs you know $60 every trip for train trips to and from the airport.
		The cost when (name of partner) use to fly back to keep her job over the Saturday and Sunday and to make sure everything was right at home…. She was doing that to keep her work going and the family going.
		Like I have ended up with heaps of tolls and fines driving through… so stressed and time is so… well you have so much going on.
		You don't know how long you're going to be there (in hospital) and you leave the ward just to get out of there to get something to eat. You spend quite a bit of money there to start with.
		You've got two lots of food and you've got the kids up here and then I've got grocery bills down there. And that's more expensive as you've got two households really.
	Caring for family and friends	Like we had someone looking after (child's name) if we weren't there and … and (being delayed to pick up child) that is another cost.
		Oh, yeah, his mobile phone bill went up the roof (increased). He was up here and I was down there.
		I would end up shouting (paying for) them lunch because I felt, you know, they were giving up there time.
		We rented a house and it only got stayed in a few months (only got stayed in a few months so you had to pay all that rent for nothing) yes.
	Diagnosis and treatment	So in relation to the cancer treatment the out of pocket which did hit us the most was when I had my stem cell and my bone marrow transplant. I had to pay for a bit of out of pocket.
		A couple of the pills were $35 and that and you get a few bottles of them and you get three bottles of them and it is $130.
		When you're an outpatient you get quite a pharmacy bill. I had quite a pharmacy bill. We were quite out of pocket. I think in the taxes it was about $1300 medical expenses.
Relying on others for	Not needing financial assistance from others [38]	No need to borrow money off family and friends.

Themes	Sub-themes	Illustrative quotes
financial support:	Aware of family members that could be called upon for financial assistance [38]	Well, I would have family I could respond to but at this stage I haven't thought about it because I haven't needed… I have family but don't need to ask.
		Yes, I have that help. I have (relatives) in mind that would gladly help me out if I was stuck … so that is a big buffer
	Received assistance from family or friends [28,32,38]	My mother gave me some money to pay for a bill and my sister said if we got desperate she would give us a loan of some money and my mother kept giving us some money when she could. I talk to my daughter and she helps us out a lot too because she paid some bills when we couldn't afford to.
		Yes, I did. My mother did all the grocery shopping. So it saved me from having to dip into my savings but it ate into her savings as well.
		Yes and we had the car payment as well which my parents paid off for us. We were well supported.
		I have had to rely on gifts from family and friends to keep from filing bankruptcy. Each year my co-pays increase and covered prescriptions decrease which put additional burdens on my budget. I have very little savings and rely on credit cards when I have unexpected expenses.
		Our daughter gave us her tips and we had friends who collected money and gave us a couple of Visa cards.
Familial or household impacts	Financial coping mechanisms [32,35,37]	Yes, you max that out (credit card) and you never catch up
		Yes, we have to use the credit card quite a lot. Usually for food, the one thing that cannot wait. You have to have it. It is a worry.
		"I had only just started at my new job. I had just been there 4 months. . . . So not any sick leave or rec leave . . . nothing. It was at a bad time, that didn't even pay my mortgage. It really ate into my savings. Because I had no sick leave or anything."
		I sold all my jewelry that my grandma gave me."
		I was earning $420 a week and I leased my car because I got it back on tax every year so I had a brand new car every year and I had money to buy things. Now I live on less than $300 a fortnight because (described hematological condition and treatment). I have had to shut down my business, move out of the house, and sell the business. I am thinking of going into bankruptcy, that is the bottom line. I have so many tiny unpaid things.
	Responsibility to others [27,40]	"The stress... I mean the financial impact... is really stressful. [And now that there are younger people being diagnosed as] young as... mid 40s... this even plays a bigger role... Because they have to stop their work and some are trying to go to work and because of the stress of the financial impacting young children and all of that"
		We have a son going to college & we cannot help with tuition.

Themes	Sub-themes	Illustrative quotes
Barriers to care due to cost [32,42]	Unable to afford care	Most of the people in village who always fall sick could not go to the hospital, this is the problem. Finance. Now, finance is really needed. We want to go to the hospital, there's no vehicle, vehicle got to pay, that is difficult. Go once can, second time can, third time cannot go already because of insufficient finance.
		"I really debated my last treatment which was photopheresis and I quite doing it because it was so expensive."
